# Effect of intraoperative lung-protective mechanical ventilation on pulmonary oxygenation function and postoperative pulmonary complications after laparoscopic radical gastrectomy

**DOI:** 10.1590/1414-431X20198523

**Published:** 2019-06-03

**Authors:** Jing Liu, Zhipeng Meng, Ran lv, Yaping Zhang, Gaojian Wang, Junran Xie

**Affiliations:** 1 Department of Anesthesia, Zhejiang University School of Medicine, Sir Run Run Shaw Hospital, Hangzhou, Zhejiang, China; 2 Department of Anesthesia, Huzhou Central Hospital, Huzhou, Zhejiang, China; 3 Department of Anesthesia, Huzhou Maternity & Child Health Care Hospital, Huzhou, Zhejiang, China

**Keywords:** Lung-protective ventilation strategy, Laparoscopic radical gastrectomy, Middle-aged and elderly people, Lung oxygenation function, Postoperative pulmonary complication

## Abstract

This study aimed to observe the effects of lung-protective ventilation (LPV) on oxygenation index (OI) and postoperative pulmonary complications (PPCs) after laparoscopic radical gastrectomy in middle-aged and elderly patients. A total of 120 patients who were scheduled to undergo laparoscopic radical gastrectomy with an expected time of >3 h were randomly divided into conventional ventilation (CV group) with tidal volume (TV) of 10 mL/kg without positive end-expiratory pressure (PEEP), and lung-protective ventilation (PV group) with 7 mL/kg TV and personal level of PEEP with regular recruitment maneuver every 30 min. Measurements of OI, modified clinical pulmonary infection score (mCPIS), and PPCs were assessed during the perioperative period. Fifty-seven patients in the CV group and 58 in the PV group participated in the data analysis. Patients in the PV group showed better pulmonary dynamic compliance, OI, and peripheral capillary oxygen saturation during and after surgery. The mCPIS was significantly lower in the PV group than in the CV group after surgery. The incidence rate of PPCs was lower in the PV group than in the CV group and the difference was significant in patients whose ventilation time was longer than 6 h in both groups. LPV during laparoscopic radical gastrectomy significantly improved pulmonary oxygenation function and reduced postoperative mCPIS and the incidence of PPCs during the early period after surgery of middle-aged and elderly patients, especially patients whose mechanical ventilation time was longer than 6 h.

## Introduction

Gastric cancer is among the most malignant tumors in China. It is a senile disease and its incidence increases with age (1). Laparoscopic radical gastrectomy is a widely used surgery and is the main method of treating gastric cancers in the world. Although the preponderance of the operation is believed to be because of its low invasiveness, good cosmetic results, and short duration of hospital stay, it produces some side effects in about 0.1–10% of patients (2). The incidence of ventilator-induced lung injury (VILI) (3–4) and pulmonary complications remains high at approximately 20–40% of upper abdominal surgeries (5), and is the main factor that negatively affects patient survival and health-care costs (6).

Lung-protective ventilation (LPV) has been reported in many studies. LPV has shown advantages in patients with healthy lungs during general anesthesia (7–8), and has demonstrated better results in patients with complications, such as lung injury in intensive care units (ICUs) and acute respiratory distress syndrome (ARDS). In recent studies, LPV has been reported to reduce VILI (9) by using some elements such as low tidal volume (TV) (10), higher positive end-expiratory pressure (PEEP) (11), and recruitment maneuver (RM) (12). PEEP, as an important component of LPV, is generally applied in clinical practice, and an unsuitable PEEP value during surgery may cause a decrease in pulmonary dynamic compliance (Cdyn) and gas exchange disorder due to the hyperinflation of lungs and/or pulmonary atelectasis in patients with normal lungs.

Therefore, the aim of this study was to titrate a suitable PEEP value in laparoscopic radical surgery for gastric cancer, to adopt a PEEP value to enact LPV, and to evaluate the effects of intraoperative LPV on lung oxygenation function and postoperative pulmonary complications (PPCs) in middle-aged and elderly patients. We hypothesized that an ideal LPV can improve lung oxygenation function compared with conventional mechanical ventilation, and hence reduce the incidence of PPCs.

## Material and Methods

### Study design

All patients gave written informed consent before inclusion. This was a double-blind, randomized, controlled study. The anesthetist in charge of the patient collected data during surgery. Ventilator settings were recorded during anesthesia, but were concealed in the case report form. The surgeon in charge of the patient was not informed of the ventilator settings. Physicians not involved in the patient's care during anesthesia and surgery carried out the postoperative evaluation, in order to preserve the double blinding. A radiologist who was not involved in the intra-operative evaluation analyzed the radiographs. Anesthesia recordings and ventilator settings during surgery were concealed from the postoperative physicians and nurses.

Participants were randomly assigned to protective ventilation (PV group) or conventional ventilation (CV group) at a ratio of 1:1. Randomization was carried out by a computer-generated blocked randomization list with 10 blocks of four patients per block, which was prepared by the Statistical Department. Allocation lists were stored in sealed, opaque numbered envelopes. Participants were included and allocated in numerical order.

Approval for the ethical aspects of the protocol of this trial was provided by the Medical Ethics Committee of Zhejiang University School of Medicine, Sir Run Run Shaw Hospital (#20180206-9). This study was retrospectively registered with the Chinese Clinical Trial Registry on August 4, 2018 and the registration number is ChiCTR1800017558.

### Inclusion and exclusion criteria

We selected patients who were scheduled for elective laparoscopic gastric cancer radical surgery under general anesthesia from August 2018 to December 2018 at Zhejiang University School of Medicine, Sir Run Run Shaw Hospital. Patients were eligible for participation if they met the following criteria: American Society of Anesthesiologists (ASA) physical status I–III patients older than 45 years of age with a body mass index (BMI) less than 30 kg/m^2^, who were candidates for elective laparoscopic gastric cancer radical surgery under general anesthesia that was expected to last more than 3 h.

The exclusion criteria were as follows: in urgent need of surgery, a history of severe restrictive pulmonary disease, a history of severe chronic obstructive pulmonary disease (GOLD grades III or IV) requiring oxygen, a history of severe or uncontrolled bronchial asthma, pulmonary infection, bronchiectasis, pulmonary metastases, use of positive pressure ventilation before surgery (e.g., continuous positive airways pressure for obstructive sleep apnea syndrome or continuous positive airway pressure, CPAP), thoracic deformities and intrathoracic diseases (such as mediastinal tumor and chest tumor), severe neuromuscular disease, the need to be transferred to ICU after surgery, severe cardiac disease defined as New York Heart Association Class III or IV, or acute coronary syndrome, or persistent ventricular tachyarrhythmia, liver cirrhosis (Child B or C), chronic renal failure with dialysis, individuals who refused to or were unable to give informed consent, and those who were participating in another interventional study.

### Standard procedures

Standard general anesthesia was performed on all participants. Participants fasted for 12 h and refrained from drinking for 8 h before surgery. After entering the operating room, under local anesthetic infiltration, a left radial arterial cannula was inserted to monitor invasive arterial blood pressure and collect blood gas samples. A right internal jugular vein catheter was inserted and standard monitoring took place. All patients accepted routine general anesthesia induction according to protocol, which contained intravenous midazolam, 0.05 mg/kg HCl, 0.6 μg/kg sufentanyl, and 0.2 mg/kg etomidate. Patients were intubated after they were administered rocuronium bromide (0.6 mg/kg). An additional 5 mg cisatracurium besylate was administered every 30 min to obtain further muscle relaxation, which was stopped at least 1 h before the end of surgical suture. Thereafter, anesthesia was kept with continuous propofol infusion (4–12 mg·kg^−1^·h^−1^), remifentanil (0.05−0.3 μg·kg^−1^·min^−1^), and sevoflurane inhalation (concentration between 1 and 3%), which was given based on the patient's bispectral index, that was maintained at 40–60. Routine intraoperative monitoring of vital signs, including invasive blood pressure, pulse oximetry, heart rate (HR), end-tidal fractions of carbon dioxide (ETCO_2_), and electrocardiograms, were performed continuously using a dedicated monitor. Intraoperative fluid and blanket warming were used to maintain core temperature above 36°C. Before the end of the operation, 8 mg ondansetron was administered in order to prevent postoperative nausea and vomiting. Postoperative intravenous analgesia was performed with 200 μg sufentanyl combined with 200 mL normal saline, the background dose was 2 mL/h, the single dose was 2 mL, and the locked time was 15 min. Postoperative analgesia lasted for 48 h after surgery to maintain a pain level <3 in a visual analogue scale (VAS) of pain from 0 to 10. If the score was above 3, intravenous 50 mg tramadol was administered for remedial analgesia. Postoperative patients were transported to the post-anesthesia care unit. After extubation, patients were oxygenated with an FiO_2_ (inspired oxygen fraction) of 0.33 through a Venturi face mask (Shanghai Xuerui Import & Export Co. Ltd., China)

The operation was performed in a pneumoperitoneum that was induced and held by applying an intra-abdominal pressure of 13–15 mmHg with room temperature CO_2_ insufflation in all patients. During the postoperative stage, all patients received routine physiotherapy (13) in line with the care standard at our hospital.

### Ventilation protocol

The patient's ideal body weight (IBW) was calculated in accordance with a predefined formula (14): 50 + 0.91 × (height (cm) − 152.4) for men and 45.5 + 0.91 × (height (cm) − 152.4) for women. Mechanical ventilation was set at the mode of volume control, TV was set to 10 mL/kg (15) based on IBW without PEEP but with FiO_2_=0.5, inspiratory to expiratory ratio was set as 1:2 in the two groups, which were carried out using the Drager Fabius anesthesia machine (Germany). Respiratory rate (starting with 12 breaths/min) was adjusted to keep normocapnia and ETCO_2_ between 35–45 mmHg. This ventilation mode was maintained for the CV group until the end of surgery, while in the PV group, 10 min after pneumoperitoneum, once a steady state had been reached, all patients were submitted to an RM using the sustained airway pressure obtained by the CPAP method and applying 30 cm H_2_O PEEP for 30 s followed by a decremental PEEP titration procedure directed by static pulmonary compliance (Cstat). During the PEEP titration procedure, PEEP was decreased from 14 cm H_2_O by 2 cm H_2_O every 4 min, until a final PEEP of 6 cm H_2_O was reached. Optimal PEEP was considered as the PEEP value resulting from the highest possible Cstat measured by the ventilator. After the PEEP titration procedure, lung-protective mechanical ventilation was performed using the optimal PEEP and TV of 7 mL/kg, and RM was repeated every 30 min during operation just before extubation (16).

### Data source and collection

The demographic characteristics including age, sex, BMI, ASA physical status, and smoking history were recorded. In both groups, intraoperative monitoring data such as mechanical ventilation time, TV, peak pressure (Ppeak), RR, crystalloids volume, colloids volume, blood loss, urine output, postoperative hospitalization days, and death rate at 30 days after surgery were also recorded. Cdyn was calculated as TV / (Ppeak -PEEP) at T1 (after intubation), T2 (10 min after pneumoperitoneum), T3 (40 min after pneumoperitoneum), and T4 (10 min after pneumoperitoneum stopped). Measurement of arterial blood gases (ABG), such as pH, PaCO_2_, and PaO_2_, were recorded at T1, T3, T5 (30 min after extubation), and T6 (the first day after operation). Peripheral capillary oxygen saturation (SPO_2_) on D2 (the second day after operation) and D5 (the fifth day after operation) were recorded; we calculated the lung oxygenation index (OI) as OI = PaO_2_ / FiO_2_ and first alveolar-arterial oxygen gradient (A-aO_2_) as A-aO_2_ = (PB - PH_2_O) × FiO_2_ - PaCO_2_ / R-PaO_2_, where PB (atmospheric pressure) was 760 mmHg, PH_2_O (saturated vapor pressure at room temperature) was 47 mmHg, and the R (respiration quotient) was 0.8.

SPO_2_ was measured in the ward air with the patient in bed. If the patient was using a nasal oxygen catheter, the catheter was removed for 10 min, and then SPO_2_ was measured after adaptation on D2 and D5. If SPO_2_ dropped to under 90% during the adaptive phase, the manipulation was stopped and the SPO_2_ measure was immediately obtained. The modified clinical pulmonary infection score (mCPIS) was calculated on D0 (the day before operation) and D2 using the modified scale as reported by Fartoukh et al. (17 [Bibr B18]) (Table 1).

**Table 1:** Scoring system (17) of the modified clinical pulmonary infection score (mCPIS).

mCPIS points	0	1	2
Tracheal secretions	Rare	Abundant	Abundant + purulent
Chest X-ray infiltrates	No infiltrates	Diffused	Localized
Temperature, ^o^C	≥36.5 and ≤38.4	≥38.5 and ≤38.9	≥39 or ≤36
Leukocytes count per mm^3^	≥4,000 and ≤11,000	<4,000 or >11,000	<4,000 or >11,000 + band forms ≥500
PAO_2_/FiO2, mmHg	>240 or ARDS		≤240 and no evidence of ARDS
Microbiology	Negative		Positive

ARDS: acute respiratory distress syndrome.

### Pathologic findings in chest radiography

Pre- and post-operative (D0 and D2) chest x-rays were performed at the bedside and were examined in a blinded way by an independent radiologist, who did not participate in this study. Pathological features were considered as the presence of at least one of the following items: increase in thickness of the interstitium, non-ventilated areas including minimal density change, atelectasis, pleural effusions, or other chest radiological alterations.

Pulmonary complications (17) were defined as the development of three or more of the following six new findings: cough, increased secretions, dyspnea, chest pain or discomfort, body temperature above 38°C, and HR above 100 beats/min. The incidence rate of PPCs between the two groups on D2 and D7 and of patients whose ventilation time was longer than 6 h in both groups on D2 and D7 were recorded.

### Primary and secondary endpoints

Our assumption was that LPV could improve oxygenation and reduce the incidence of PPCs. The primary endpoints were the change in pulmonary oxygenation including OI and A-aO_2_ during the pre- and postoperative period. The secondary endpoints were the changes in pulmonary Cdyn during operation, mCPIS, and the incidence of PPCs.

### Statistical analysis

The sample size calculation formula (19) was: n = [(Zα /2 + Zβ) 2 × 2 (standard deviation) 2 / (µ1 - µ2) 2], where n = sample size required in each group, μ1 = mean of OI in the PV group, μ2 = mean of OI in the CV group, μ1 - μ2 = clinically significant difference, Zα/2: 5% level of significance (1.96), Zβ: 95% power (1.96), and standard deviation = 1.195. We designed a pilot study with 24 patients and detected a significant difference in Cdyn and OI between groups, in which μ1 was measured as 1.05 and μ2 as 1.25. Therefore, n was equal to 60 for each group, which gave us a total sample size of 120. Statistical analysis was carried out using SPSS version 17 (IBM, USA). Quantitative data are reported as means±SD. Qualitative data are reported as frequencies and percentages. Quantitative variables were compared between two groups using Student's t-test. The comparison of qualitative variables between two groups was made with the chi-squared test. In all cases, the statistically significant level was set at a P value <0.05.

## Results

The 120 patients were divided into the two groups using block randomization with Stata software (StataCorp, USA). Three patients assigned to the CV group and two patients assigned to the PV group were eliminated because of alteration of the operation plan to laparotomy surgery. Finally, 115 patients were enrolled, with 57 in the CV group and 58 in the PV group. Baseline characteristics had no significant differences between the two groups (Table 2).

**Table 2:** Baseline characteristics of patients.

	CV group (n=57)	PV group (n=58)	P value
Gender F/M (n)	31/26	32/26	0.93
Age, years (means±SD)	66.13±9.12	63.20±8.31	0.07
BMI, kg/m^2^ (means±SD)	23.27±2.95	22.45±2.10	0.08
ASA I, n (%)	7 (12.28)	8 (13.79)	0.81
ASA II, n (%)	40 (70.18)	39 (67.24)	0.73
ASA III, n (%)	10 (17.54)	11 (18.97)	0.84
History of hypertension, n (%)	10 (17.54)	12 (20.69)	0.67
History of cardiopathy, n (%)	5 (8.77%)	3 (5.17%)	0.45
Smoking history, n (%)	8 (14.04%)	9 (15.52%)	0.82

PV: protective ventilation; CV: conventional ventilation; BMI: body mass index; ASA: American Society of Anesthesiologists. Data were analyzed by chi-squared test or *t*-test.

TV was lower and RR was higher in the PV group compared with the CV group, whereas there was no statistically significant difference between the two groups with regard to intraoperative crystalloids volume, colloids volume, blood loss, urine output, and postoperative length of stay in hospital (Table 3).

**Table 3:** Intraoperative monitoring of patients treated with protective ventilation (PV) and conventional ventilation (CV).

	CV group (n=57)	PV group (n=58)	P value
Operation time (min)	301.34±54.27	298.95±65.76	0.21
Mechanical ventilation time (min)	320.77±65.22	323.25±70.83	0.85
Tidal volume, mL	535.64±59.18	440.64±55.37	<0.001
RR, breaths/min	12.59±0.93	13.54±1.13	<0.001
Crystalloids volume, mL	1608.97±391.39	1648.65±341.35	0.63
Colloids volume, mL	519.23±87.42	543.705±123.59	0.31
Blood loss, mL	82.82±57.38	79.25±47.87	0.76
Urine output, mL	382.05±171.15	346.25±134.81	0.21
Postoperative hospital stay, days	11.2±3.3	12.6±4.1	0.88
Death rate at 30 days after surgery, %	0	0	1

RR: respiratory rate. Data are reported as means±SD (chi-squared test or *t*-test).

**Figure 1:**
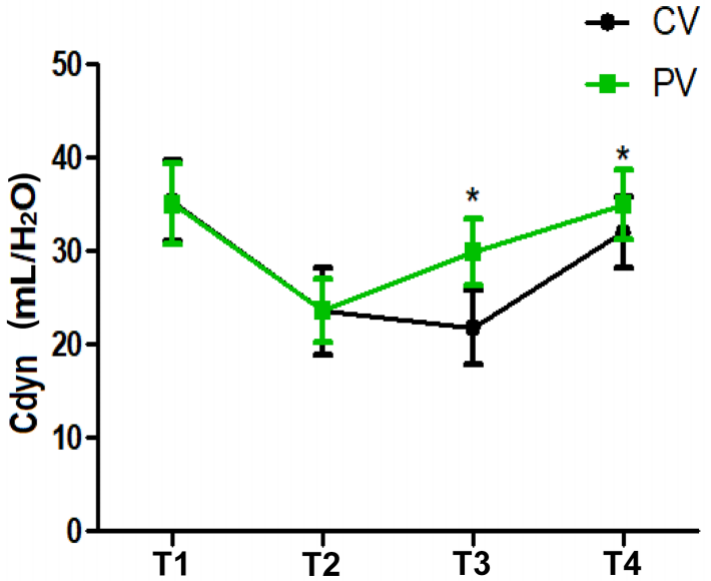
Intraoperative pulmonary dynamic compliance (Cdyn) at T1 (after intubation), T2 (10 min after pneumoperitoneum), T3 (40 min after pneumoperitoneum), and T4 (10 min after pneumoperitoneum stopped) in patients treated with protective ventilation (PV) and conventional ventilation (CV). Data are reported as means±SD. *P<0.05 compared with the CV group (*t*-test).

Compared with T1, Cdyn decreased at T2 in both groups (P<0.05); compared with the CV group, Cdyn clearly increased at T3-T4 in the PV group (P<0.05) (Figure 1).

**Figure 2:**
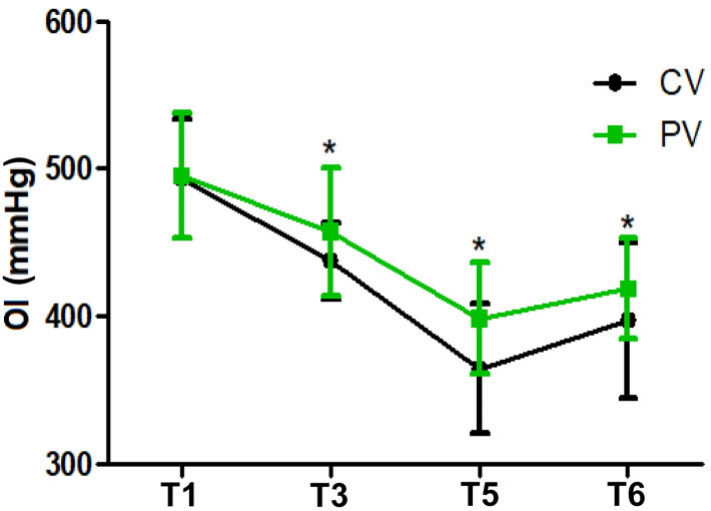
Oxygenation index (OI) at T1 (operative, after intubation), T3 (40 min after pneumoperitoneum), and postoperative periods T5 (30 min after extubation) and T6 (one day after surgery) in patients treated with protective ventilation (PV) and conventional ventilation (CV). Data are reported as means±SD. *P<0.05 compared with the CV group (*t*-test).

**Figure 3:**
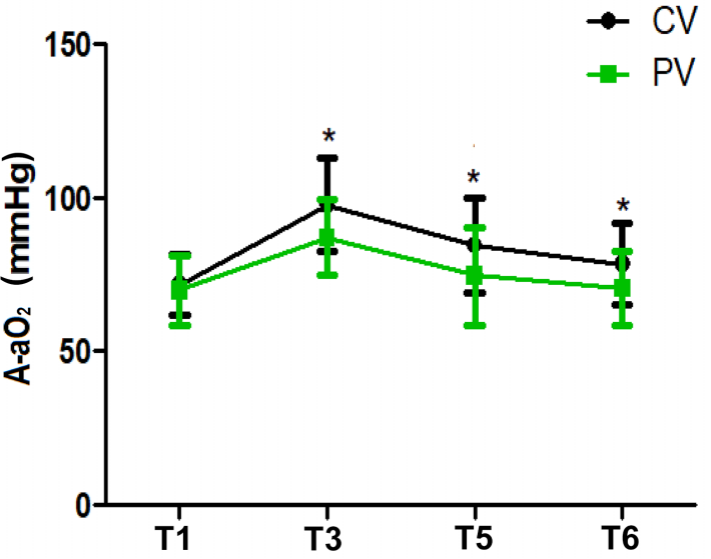
Alveolar-arterial oxygen gradient (A-aO_2_) at T1 (operative, after intubation), T3 (40 min after pneumoperitoneum), and postoperative periods T5 (30 min after extubation) and T6 (one day after surgery) in patients treated with protective ventilation (PV) and conventional ventilation (CV). Data are reported as means±SD. *P<0.05 compared with the CV group (*t*-test).

**Figure 4:**
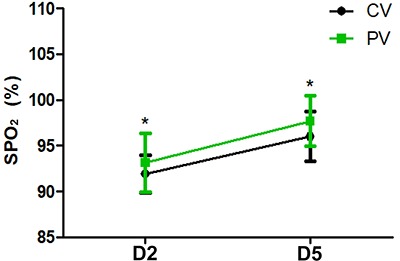
Peripheral capillary oxygen saturation (SPO_2_) at day two (D2) and day 5 (D5) after surgery in patients treated with protective ventilation (PV) and conventional ventilation (CV). Data are reported as means±SD. *P<0.05 compared with the CV group (*t*-test).

Compared with T1, the OI value was lower at T3, T5, and T6 (P<0. 05), while in the PV group the OI value was higher than that of the CV group at T3, T5, and T6 (P<0. 05) (Figure 2). Compared with T1, the A-aO_2_ was elevated at T3, T5, and T6 (P<0.05), while in the PV group the A-aO_2_ was lower than that of the CV group at T3, T5, and T6 (P<0.05) (Figure 3). In the PV group, SPO_2_ was higher than that of the CV group on D2 and D5 (P<0.05) (Figure 4).

**Figure 5:**
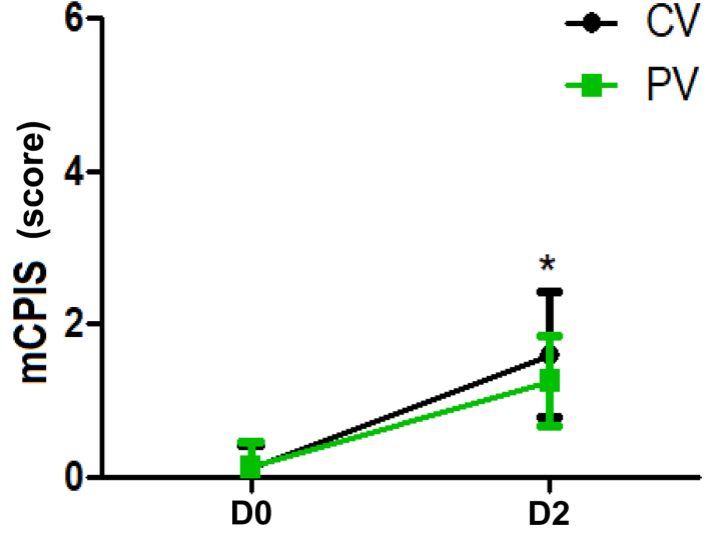
Results of modified clinical pulmonary infection score (mCPIS) on the day of the surgery (D0) and day 2 (D2) after surgery in patients treated with protective ventilation (PV) and conventional ventilation (CV). Data are reported as means±SD. *P<0.05 compared with the CV group (chi-squared test).

**Figure 6:**
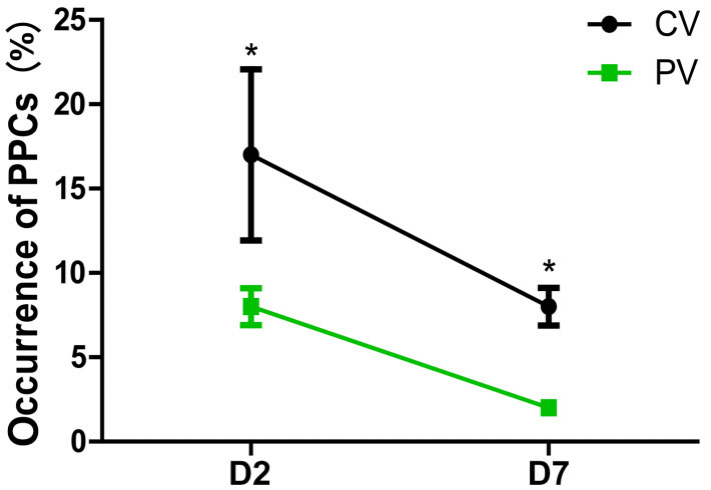
Occurrence of postoperative pulmonary complications (PPCs) on day 2 (D2) and day 7 (D7) after surgery in patients treated with protective ventilation (PV) and conventional ventilation (CV). Data are reported as means±SD. *P<0.05 compared with the CV group (chi-squared test).

**Figure 7:**
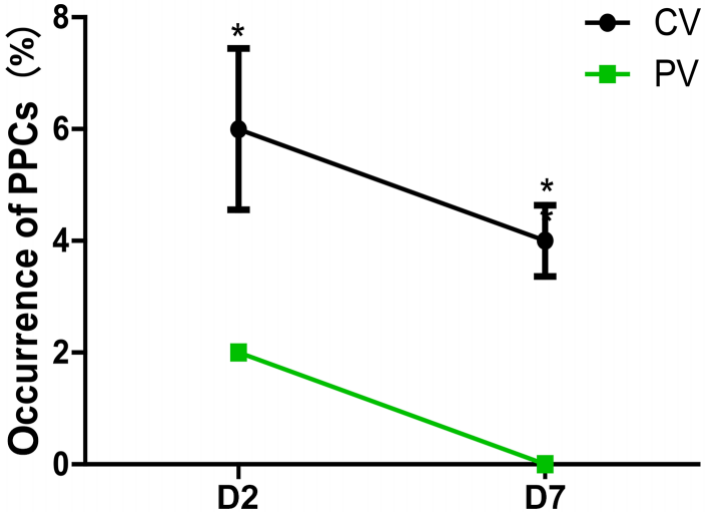
Incidence of postoperative pulmonary complications (PPCs) of patients with ventilation time longer than 6 h in patients treated with protective ventilation (PV) and conventional ventilation (CV) on day 2 (D2) and day 7 (D7) after surgery. Data are reported as means±SD. *P<0.05 compared with the CV group (chi-squared test).

On D2, the mCPIS was lower in the PV group compared with that of the CV group (P<0. 05) (Figure 5). The incidence of PPCs was lower in the PV group compared with the CV group (Figure 6). Similar results were found for patients with ventilation time longer than 6 h on D2 and D7 (Figure 7).

## Discussion

The main findings of this study were: 1) PV improved the outcomes by significantly increasing respiratory system compliance, pulmonary oxygenation function, and peripheral oxygen saturation during the perioperative and postoperative period, and the beneficial effects persisted after extubation; 2) PV decreased mCPIS on D2 and reduced the occurrence rate of PPCs on D2 and D7, especially in patients with ventilation time longer than 6 h.

CO_2_ pneumoperitoneum can make the ventilated lung more fragile and increase the risk of developing VILI. Inappropriate mechanical ventilation settings during general anesthesia could aggravate and even initiate lung damage in patients with normal lungs at the onset of ventilation which may lead to VILI (3,4). The majority of elective laparoscopic radical gastrectomy procedures last more than 3 h with general anesthesia; notably, both laparoscopic abdominal surgery and longer duration of anesthesia have been reported as potential risk factors for higher incidence of PPCs. LPV has been reported to decrease the death rate of moderate to severe forms of ARDS from almost 70 to 40% (20). LPV has also been proven to enhance postoperative outcomes and reduce the duration of hospital stay in numerous studies (15,21,22). However, a recent experimental research concluded that inadequate PEEP is problematic, as it may lead to lung injury, decrease pulmonary compliance and increase inflammation in normal lungs (23). Properly adjusted PEEP may have significant protective effects, whereas inadequate PEEP may promote pulmonary atelectasis and/or hyperinflation of dependent lung tissue. For example, a high PEEP might result in more hyper-distension than collapse whereas low PEEP might result in more collapse than hyper-distension. Therefore, in order for PEEP to be effective, it must be personalized to the individual lung physiology of each patient. However, the efficacy of using an optimal PEEP level has not been studied thoroughly in laparoscopic radical gastrectomy.

Our research aimed to find the ideal PEEP for patients and assess whether LPV could boost lung oxygenation function and reduce the occurrence of PPCs in patients undergoing laparoscopic gastric cancer radical surgery.

The PEEP titration procedure demonstrated not only that CO_2_ pneumoperitoneum promoted massive lung collapse, but also that an improvement in lung function could be achieved by applying LPV, with long-lasting effects after surgery and minimal side effects. Titrating PEEP and performing this PEEP with regular RM in the course of mechanical ventilation also had benefits as demonstrated by near-term clinical tests (8,24). We deliberately chose to combine lower TV with individualized PEEP level as well as RM to identify a ventilation strategy aimed at keeping the lung open during general anesthesia for surgery. Our data suggested that optimal PEEP made a marked improvement in arterial oxygenation and provided better peripheral oxygen saturation in the PV group during and post-operation, which was in line with the research of Severgnini et al. (25).

Oxygenation was studied while the patients were breathing in air, in a seated position, after 10 min of adaptation. This allowed avoiding any possible influence of different inspiratory oxygen fractions on the arterial oxygenation. Similarly, we observed a lower percentage of patients with peripheral oxygen saturation levels less than 90% in air in the PV group compared to the CV group (1.7 *vs* 17.5% respectively, P<0.05). Applying an individualized lung-protective ventilatory strategy during general anesthesia may improve the gas exchange and prevent the development of lung collapse and atelectasis not only intraoperatively but also in the postoperative period in our trial.

The mCPIS was calculated by a modified scale as described by Fartoukh et al. (17). The scale is a comprehensive clinical, imageology system developed to evaluate the severity and prognosis of infection, and had great clinical application value for the evaluation of early lung infection. In our trial, the mCPIS was calculated only on postoperative day 2 and we found that the PV group was associated with a statistically significant reduction in mCPIS compared with the CV group. The result showed that LPV could decrease the mCPIS and pulmonary infection. LPV had a good preventive effect on PPCs in patients undergoing laparoscopic gastric cancer radical surgery.

PPCs caused by mechanical ventilation usually occur within 5–7 days after surgery. In order to exclude pulmonary complications caused by insufficient postoperative respiratory exercise or postoperative anastomotic fistula, the occurrence of PPCs was analyzed by an experienced physician during the first 7 postoperative days in this study. The analysis showed that the incidence of PPCs in the PV group was significantly lower than in the CV group. This result was consistent with previous studies: Serpa et al. (26) meta-analysis of more than 2000 patients undergoing general anesthesia found that, compared with the traditional TV ventilation mode, the protective pulmonary ventilation mode can effectively prevent the occurrence of PPCs.

Surprisingly, we also found that for patients with ventilation time longer than 6 h in both groups, the incidence of PPCs in the PV group was remarkably lower than that in the CV group. This implied that protective ventilation might be beneficial for patients with the ventilation time longer than 6 h. However, as the size of the samples was small, our study was unable to demonstrate a notable decrease in the incidence of long-term major pulmonary complications.

To the best of our knowledge, our study was the first to find a moderate PEEP value and then investigate the effect of LPV patients undergoing laparoscopic radical gastrectomy. However, this study has limitations that ought to be mentioned. We selected the combination of lower TV with a moderate PEEP level and regular RM to confirm a ventilation strategy with the purpose of maintaining an open lung and a better lung oxygenation function during the course of mechanical ventilation, and the strategy may be beneficial during the postoperative period. We did not concentrate on the effects of ventilation strategies on major PPCs because of the small sample. The mCPIS uses the evaluation of chest x-ray, which may underestimate the existence of atelectasis and lung morphology transformations compared to computed tomography (CT) (27). However, CT is hard to acquire in our hospital due to economic and ethical factors.

## Conclusion

In conclusion, LPV with lower TV, moderate PEEP, and regular RM during the course of anesthesia can significantly improve pulmonary oxygenation function and reduce the incidence of PPCs, especially in patients with a ventilation time longer than 6 h. Larger sample sizes and long-term evaluation are recommended for future studies.
